# Serratamolide is a Hemolytic Factor Produced by *Serratia marcescens*

**DOI:** 10.1371/journal.pone.0036398

**Published:** 2012-05-16

**Authors:** Robert M. Q. Shanks, Nicholas A. Stella, Roni M. Lahr, Shaoru Wang, Tara I. Veverka, Regis P. Kowalski, Xinyu Liu

**Affiliations:** 1 Charles T. Campbell Laboratory of Ophthalmic Microbiology, Department of Ophthalmology, University of Pittsburgh Medical Center, Pittsburgh, Pennsylvania, United States of America; 2 Department of Chemistry, University of Pittsburgh, Pittsburgh, Pennsylvania, United States of America; University of Birmingham, United Kingdom

## Abstract

Serratia marcescens is a common contaminant of contact lens cases and lenses. Hemolytic factors of S. marcescens contribute to the virulence of this opportunistic bacterial pathogen. We took advantage of an observed hyper-hemolytic phenotype of crp mutants to investigate mechanisms of hemolysis. A genetic screen revealed that swrW is necessary for the hyper-hemolysis phenotype of crp mutants. The swrW gene is required for biosynthesis of the biosurfactant serratamolide, previously shown to be a broad-spectrum antibiotic and to contribute to swarming motility. Multicopy expression of swrW or mutation of the hexS transcription factor gene, a known inhibitor of swrW expression, led to an increase in hemolysis. Surfactant zones and expression from an swrW-transcriptional reporter were elevated in a crp mutant compared to the wild type. Purified serratamolide was hemolytic to sheep and murine red blood cells and cytotoxic to human airway and corneal limbal epithelial cells in vitro. The swrW gene was found in the majority of contact lens isolates tested. Genetic and biochemical analysis implicate the biosurfactant serratamolide as a hemolysin. This novel hemolysin may contribute to irritation and infections associated with contact lens use.

## Introduction

*Serratia marcescens* is a nosocomial pathogen [1], [2], [3], a common contaminant of contact lens cases and is associated with a number of ocular conditions including keratitis, conjunctivitis, and contact lens acute red eye (CLARE) [4]. Hemolysins are important virulence factors for a wide range of Gram-negative and Gram-positive organisms [5], [6], [7], [8], [9]. Known *S. marcescens* hemolytic exoenzymes are ShlA and PhlA. ShlA is a key virulence factor and a pore-forming hemolysin [10], [11], whereas PhlA is phospholipase, one of whose cleavage products is lysophospholipid, a surfactact that can lyse red blood cells [12].

Regulators of the *shlA* hemolysin gene include the FlhDC flagellar biosynthesis regulator and RssAB, a two component transcriptional regulator [11]. RssAB is a negative regulator of *flhDC* expression, whereas FlhDC is a positive regulator of the *shlA* hemolysin operon, *shlBA* [11]. It was also shown that the cyclic nucleotide cAMP, the adenylate cyclase (CyaA) that generates cAMP, and the cAMP-receptor protein transcription factor (CRP) cAMP-CRP positively regulate FlhDC [13], [14]. The *phlA* gene is also directly regulated by FlhDC and catabolite repression [15], [16]. Therefore, it would be predicted that *crp* mutants should have reduced hemolytic activity through a reduction of both *shlA* and *phlA* expression. Unexpectedly, we observed that *crp* mutants exhibited increased levels of hemolytic activity, suggesting another mechanism of hemolysis. Here we used a genetic approach to gain insight into the mechanism of hemolysis exhibited by *crp* mutants. Genetic and biochemical analysis in this study support the model that the biosurfactant serratamolide is a hemolysin.

## Results

### Mutations in *crp* and *cyaA* Lead to an Increase in Secreted Hemolytic Activity that is Independent of known Hemolytic Agents ShlA and PhlA

**Figure 1:**
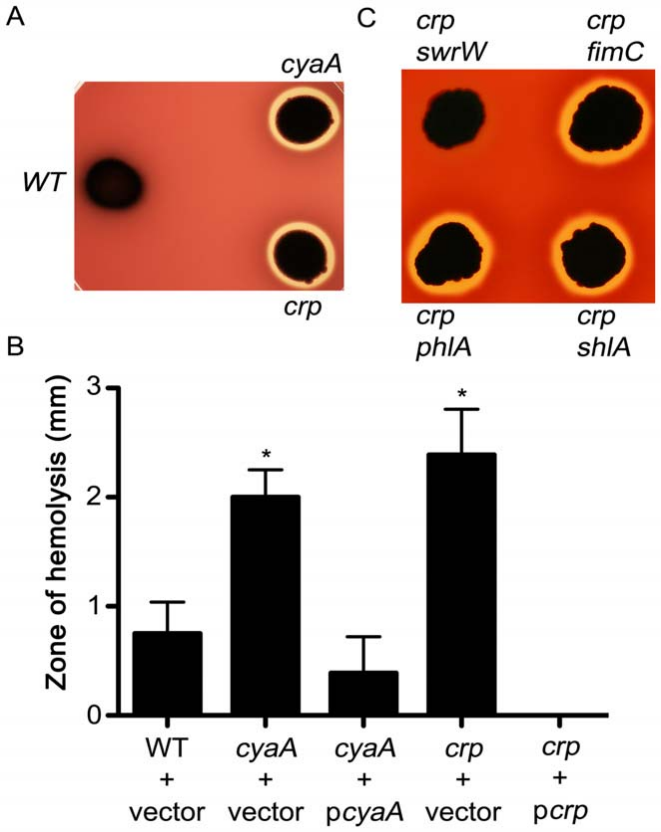
Hyper-hemolysis phenotype of *crp* mutants and genetic analysis. A. *S. marcescens* with mutations in *cyaA* or *crp* exhibit a hyper-hemolysis phenotype compared to the isogenic WT strain on TSA agar with 5% sheep red blood cells at 48 hours. **B.** Complementation of *cyaA* and *crp* hyper-hemolysis phenotypes with wild-type copies of the respective genes on multicopy plasmids (p*cyaA* = pMQ157, p*crp* = pMQ166). Asterisks indicate significantly larger zones (72h) than the WT (p<0.05, ANOVA with Tukey’s post-test). **C**. Double mutant hemolysis phenotypes show that expected hemolysin genes, *phlA* and *shlA* are not required for the *crp* mutant phenotype. The *swrW* gene is required, and a control for insertion mutagenesis (*fimC*) is included.

Previously *cyaA* and *crp* null mutants were characterized for exhibiting elevated fimbriae and prodigiosin production [17]. Here we describe a novel hemolysis phenotype for these mutants. The *cyaA* and *crp* mutant strains exhibited dramatically increased zones of hemolysis on blood agar plates compared to the parental, wild-type (WT) strain CMS376 [18], that produces small zones of hemolysis after several days of incubation at 30°C (Fig 1A). The hyper-hemolytic phenotype could be reversed by returning the wild-type *cyaA* and *crp* genes, respectively, on a multicopy plasmid (Figure 1B). From this point onward, we focused on *crp* mutants, for simplicity.

We tested whether *S. marcescens* exoenzymes, ShlA and PhlA, were required for increased extracellular hemolysis produced by *crp* mutants. If one of these enzymes is required for the increased hemolysis seen in *crp* mutants, then mutation of *shlA* or *phlA* should eliminate the hyper-hemolysis phenotype of the *crp* mutants. However, disruption of the *shlA* and *phlA* genes did not decrease the large hemolytic zones of *crp* mutant, suggesting that another hemolysis-promoting factor was involved (Fig 1C). Integration of a similar plasmid at *fimC* was used as a plasmid integration control, and had no impact on hemolysis (Fig 1C).

A potential hemolytic role for serralysin and prodigiosin, a cytotoxic protease [19]–[20] and membrane-associated pigment respectively, were similarly disproved, as *crp prtS* and *crp pigB* double mutants still exhibit high levels of hemolysis (data not shown).

### Suppressor Analysis of the *crp* Hyper-hemolysis Phenotype Implicates Serrawettin as a Hemolytic Factor

**Figure 2:**
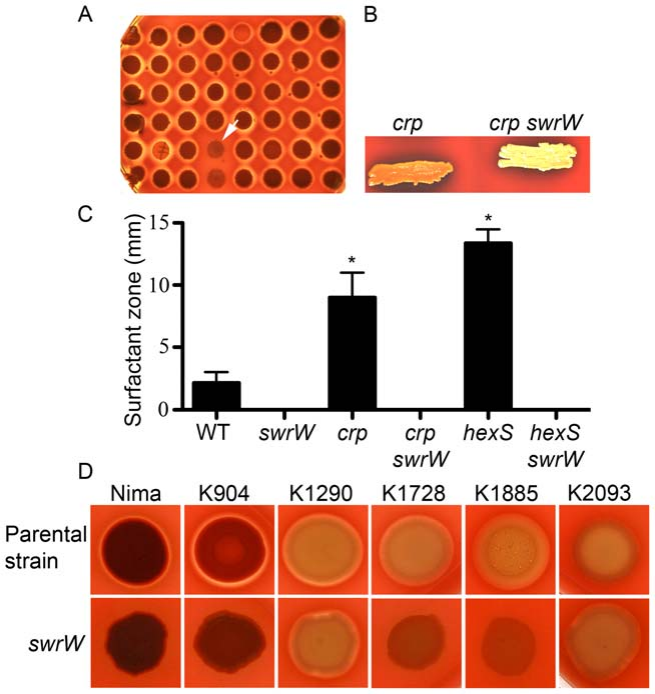
Isolation of *swrW* and its role in surfactant production and hemolysis. A. Sample genetic screen plate shows *crp* mutants with random transposon insertions. The white arrow indicates a colony deficient in secreted hemolysis production with a transposon insertion that mapped to the *swrW* gene. This image is illuminated from the back, so that the gold surface coloration is not apparent. **B.** Surface coloration of *crp swrW* double mutants is metallic gold compared to the red-orange color of the *crp* mutant. **C.** Surfactant zones (mm) measured from the colony to the maximum extent of the surfactant zone (n≥4 per genotype). Asterisk represents a statistically significant increase in surfactant zone compared to the WT (p<0.05) by ANOVA with Tukey’s post-test. **D.** Mutation of *swrW* reduced or eliminated the ability of laboratory strain Nima and three of five clinical keratitis isolates to make zones of hemolysis on blood agar plates. Representative images from reproducible experiments are shown.

To determine the mechanism of hyper-hemolysis, a suppressor analysis approach was taken using random transposon mutagenesis [18]. Multiple mutations that inhibited secreted hemolysis production were identified in a *crp* mutant background (Fig 2A). The transposon insertion sites from these hemolysis deficient isolates were scattered along the length of the *swrW* gene (base pair 821, 831, 1396, 2585, and 3078). Strikingly, mutation of the *swrW* gene led to an unreported metallic gold color on the surface of colonies (Fig. 2B). In Figure 2B the image is illuminated from the top to depict the golden coloration, whereas the rest of the images of blood agar plates are illuminated from below to exhibit the zones of hemolysis. The *swrW* gene was previously implicated in production of serratamolide, a dilactone biosurfactant with antimicrobial activity, also known as serrawettin W1 [21], [22], [23]. Serratamolide was previously shown to be required for surface swarming motility of some strains of *S. marcescens* [24]. Other surface wetting agents made by *S. marcescens* are the chemically distinct serrawettin W2 and W3, which are both larger cyclic-peptides, composed of five amino acids and a single acyl side chain [24], [25], [26].

Zones of surfactant were visible on top of agar plates surrounding colonies. We measured this zone and found that it was significantly larger (p<0.05) around *crp* mutants (9.0±2.0 mm) compared to the WT (2.2±0.9 mm) (Fig 2C). Mutation of the *swrW* gene in the WT and *crp* background completely eliminated this zone (Fig 2C). These data suggest that the surfactant zone is serratamolide and that *crp* mutants produce more of it.

**Figure 3:**
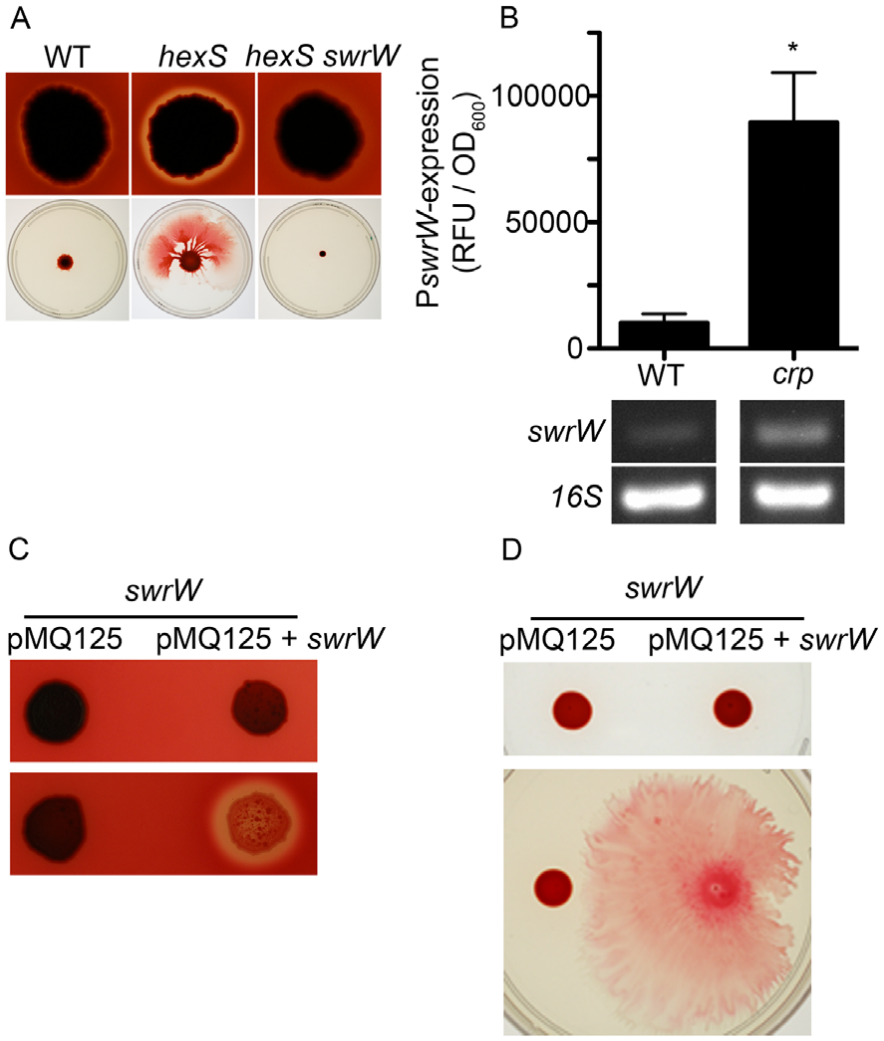
Genetic evidence that serratamolide mediates hemolysis. A. Hemolysis and swarming by a mutant known to have elevated serratamolide production (*hexS*) is increased, and these phenotypes require SwrW. **B.** Elevated expression of a *swrW* promoter reporter in the *crp* mutant. Top, expression measured using a plasmid based-*tdtomato* reporter construct at t = 20 hrs. Asterisk indicates statistical significance (p<0.05) by the Student’s T-test. A representative experiment is shown (n = 4). Error bars indicate one standard deviation. Bottom, semi-quantitative RT-PCR analysis of RNA from WT and Δ*crp* mutant strains measured relative expression of *swrW* and internal standard 16S RNA from stationary phase cultures (OD_600_ = ∼3.5). **C.** Arabinose-inducible expression of the *swrW* gene in an *swrW* transposon mutant strain restores hemolysis. **D.** Swarming motility defect of the *swrW* mutant is restored by induced expression of the *swrW* gene.

To confirm the above observations, we directly mutagenized the *swrW* gene. An internal fragment of *swrW* was cloned into suicide-promoter probe plasmid, pStvZ3, and introduced into WT and *crp* mutant strains by conjugation, as previously described [14]. The *swrW::*pStvZ3 and *crp swrW::*pStvZ3 mutant strains did not produce zones of surfactant (Fig 2C) or hemolysis (Fig 3C, and data not shown). Mutation of *swrW* in the laboratory strain Nima and a pigmented clinical isolate K904 led to a similar metallic gold colony color and deficiency in hemolysis on blood agar plates, indicating that the observed phenotypes are not restricted to the CMS376 laboratory strain (Fig 2D and data not shown).

If *swrW* expression positively impacts hemolysis, then a mutant strain with elevated levels of *swrW* expression would be expected to be more hemolytic. It has been previously reported that the LysR-family transcription factor, HexS, directly inhibits transcription of *swrW* [27]. We mutated the *hexS* gene to test whether it would confer a hyper-hemolysis phenotype, and found that *hexS* mutants exhibited elevated hemolytic zones similar to the *crp* mutant (Fig 3A). A *hexS swrW* double mutant was non-hemolytic, indicating that the hyper-hemolytic phenotype of *hexS* mutants depends upon *swrW* (Fig 3A). As with the *crp* mutant, *hexS* mutants exhibited significantly larger (p<0.05) surface surfactant zones (13.4±1.1 mm) than the WT, whereas the *hexS swrW* mutant exhibited no surfactant zones (Fig 2C).

Consistent with serratamalide promoting swarming motility, we tested whether *hexS* mutants would produce larger zones of swarming. Whereas, the WT strain is competent at swarming, the Δ*hexS* mutant swarmed earlier and to a greater extent (Figure 3A and data not shown). This swarming phenotype was eliminated in the *hexS swrW* double mutant indicating that the *hexS* hyper-swarming phenotype is serratamolide dependent (Figure 3A).

To test the prediction that *swrW* is more highly expressed in a *crp* mutant background, the *swrW* promoter (P*swrW*) was cloned in front of the *tdtomato* reporter gene on a pBBR1-based plasmid (Fig 3B). This plasmid was placed in WT and *crp* mutant strains and fluorescence was measured as a function of culture density. We observed elevated levels of red-fluorescence in the *crp* mutant compared to the WT strain. Strains bearing a control plasmid without tdtomato exhibited negligible fluorescence (data not shown). A similar result was observed with semi-quantitative RT-PCR analysis of the WT and Δ*crp* mutant, where *swrW* transcript was more abundant in Δ*crp* mutant RNA preparations relative to those from the WT (Figure 3B). These analyses are consistent with a model where cAMP-CRP negatively regulates *swrW* gene expression in a direct or indirect manner.

### Complementation of the *swrW* Mutant Hemolysis and Swarming Phenotypes

Because the *swrW* gene is not present in the sequenced Db11 strain of *S. marcescens*, we do not know its genetic context, so it is possible that some of the mutant phenotypes are due to polar effects on adjacent genes. To ensure that the *swrW* mutations were responsible for the mutant phenotypes, we cloned the full-length *swrW* gene and placed it under transcriptional control of the *E. coli P_BAD_* promoter in vector pMQ125 [28] to generate the pMQ367 plasmid. The *swrW::*pStvZ3 mutant bearing the *P_BAD_ -swrW* plasmid was rescued with respect to swarming motility and exhibited a hyper-hemolysis phenotype when L-arabinose was supplemented in the medium to induce *swrW* expression, but not in medium without L-arabinose (Fig 3C–D). As expected, the *swrW::*pStvZ3 mutant bearing the empty vector, pMQ125, did not swarm or exhibit zones of hemolysis even with L-arabinose induction (Fig 3C–D). As a control for the effect of L-arabinose on hemolysis and swarming in general, it was also determined that L-arabinose did not restore swarming or hemolysis to the *swrW* mutant without the *P_BAD_ −swrW* construct (data not shown). These data indicate that mutation of *swrW* rather than a polar effect on an adjacent gene or another mutation is responsible for the *swrW* mutant phenotypes.

### Serratamolide can act as a Hemolysin

**Figure 4:**
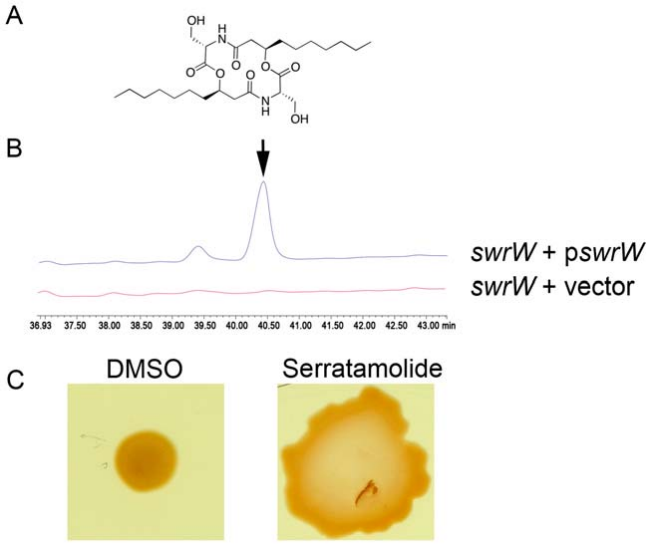
Serratamolide purification and verification of biological activity. A. Structure of serratamolide. **B.** HPLC trace of spent supernatants from a *swrW* mutant with either an empty vector (*swrW*+vector) or a *swrW* expression plasmid (*swrW*+p*swrW*). The expected peak for serratamolide is indicated by an arrow. **C.** Swarming motility of an *swrW* mutant treated with DMSO or purified serratamolide. This shows that the purified compound restores swarming motility as expected.

Serratamolide, a cyclic and aliphatic aminolipid [21], [22] (Fig 4A), was purified to verify its role as a hemolysin. Comparative profiling of secreted metabolites by the *S. marcescens* WT, and the *swrW* mutant strain with both the empty vector and expressing *swrW* from p*swrW* (pMQ367), using HPLC and LC-MS, clearly indicated that the key difference lies at a metabolite fraction with m/z at 515.5 that corresponds to serratamolide (Fig 4B and data not shown). To unequivocally assign its molecular identity, we isolated the corresponding fraction by preparative HPLC and confirmed its structural identity by HR-MS and NMR analysis. All spectral data were in accordance with previous reports on serratamolide [29] (data not shown). Furthermore, the purified serratamolide was able to restore swarming activity to an *swrW* mutant strain, providing biological evidence that the purified compound is serratamolide (Fig 4C).

**Figure 5:**
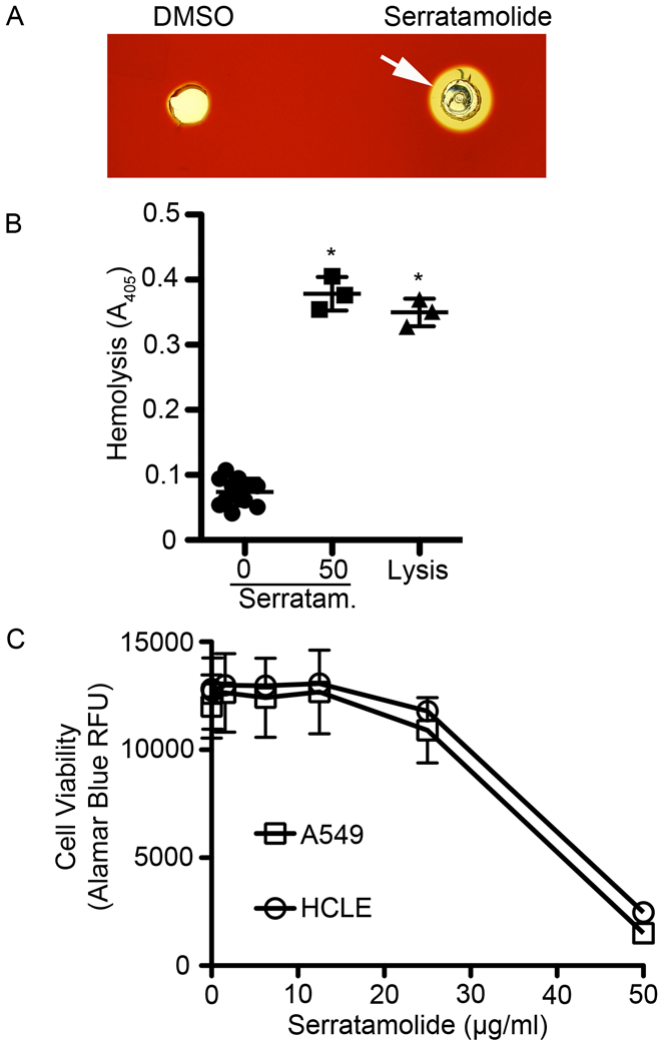
Serratamolide is hemolytic to erythrocytes and cytotoxic to epithelial cells *in vitro*. A. Hemolysis of DMSO and serratamolide (1 mg/ml) to sheep red blood cells. Wells were cut into a TSA+sheep blood agar plate, and DMSO or serratamolide was added to the well and incubated for 24 hours. White arrow indicates zone of hemolysis around serratamolide treated well. **B.** Hemolysis of murine red blood cells in solution by serratamolide (µg/ml) incubated for 10 seconds. Distilled water was used as a complete lysis control (Lysis). A representative experiment is shown. Error bars indicate one standard deviation. **C.** Cytotoxicity to immortalized human airway (A549) and corneal (HCLE) epithelial cells was measured using alamar blue fluorescence that provided a positive output for viability of cells. The average of eight independent replicates is shown for each cell line from two separate experiments. Error bars indicate one standard deviation.

Purified serratamolide (1 mg/ml) placed in wells in blood agar plates created clear zones of hemolysis, unlike the DMSO mock control (Fig 5A). In addition to rabbit erythrocytes, we observed that serratamolide at a concentration of 20.8 µg/ml completely lysed mouse erythrocytes in solution in less than 10 seconds (Fig 5B).

To test whether the cytotoxic effect of serratamolide is specific to red blood cells, we tested whether serratamolide reduced the viability to immortalized human airway epithelial cell (A549) and human corneal limbal epithelial cell (HCLE) monolayers using a fluorescent viability dye. We observed a dose-dependent cytotoxic effect at a concentration above 12.5 µg/ml (Fig 5C). At the maximum serratamolide concentration of 50 µg/ml the fluorescent viability value of HCLE cells was 1821±432, significantly less than 10483±232 for mock treated cells (p<0.05, Student’s T-test), and similar to 897±5 for detergent lysed cell layers. Similar, significant results were observed for A549 cells (Fig 5C). Fluorescent values for serratamolide treated cells can be compared to those controls values to calculate a % cytotoxicity metric. Serratamolide at 50 µg/ml caused 88.0±2.5% cytotoxicity to A549 lung cells and 95.4±4.0% cytotoxicity to HCLE ocular cells, signifying that serratamolide treatment is toxic to human epithelial cell lines.

### Contact Lens Associated Isolates are More Likely to have *swrW* than Keratitis Isolates

We tested a library of clinical isolates from the Charles T. Campbell laboratory of Ophthalmic Microbiology from keratitis patients for the presence of the *swrW* gene. PCR was used to probe for the presence of the *swrW* gene in the chromosome of these strains. *Staphylococcus aureus* chromosomal DNA was used as a negative control, and the *S. marcescens oxyR* gene was used as a positive PCR target to ensure DNA quality. Out of all 63 isolates, a total of 22 (34.5%) exhibited PCR amplicons consistent with the *swrW* gene.

Of the *swrW* positive clinical isolates, 9 out of 22 (40.1%) tested positive for hemolysis on blood agar plates. To test whether their hemolytic phenotypes were *swrW* dependent, the *swrW* gene was mutated in five of the clinical isolates. Mutation of *swrW* in the five different clinical isolates conferred a loss of swarming motility (data not shown), and three of these five also became deficient in the ability to create zones of hemolysis on blood agar plates (Fig 2D).

## Discussion

The data presented here indicate that the bio-surfactant serratamolide can act as a novel *S. marcescens* hemolysin, and that the non-ribosomal peptide synthetase SwrW is necessary for hemolysis in some clinical and laboratory strains. This conclusion is based upon the genetic data that hemolysis is eliminated in *swrW* mutants and elevated in *crp* and *hexS* mutants that over-express *swrW*. Biochemical data indicate that purified serratamolide is sufficient to lyse red blood cells and damage epithelial cells *in vitro*.

Genetic data suggests that serratamolide production is regulated by cAMP-CRP in strain CMS376, namely that surfactant zones are increased in a *crp* mutant and hemolysis is increased in mutant strains with altered ability to respond to or make cAMP (*crp* and *cyaA*). Since the cAMP-CRP pathway is well known to regulate genes in response to the nutritional environment of the cell, this may indicate that serratamolide plays a role in a bacterium’s ability to acquire or compete for nutrients. Consistent with the role of serratamolide in competition, it has been shown that serratamolide has antimicrobial activity against both prokaryotes and fungi [22], [29], and that swarming motility, which requires biosurfactants such as serratamolide, confers resistance to antibiotics [30]. Another role for serratamolide was suggested by Barr-Ness and colleagues [31]. They showed that mutant strains deficient in serratamolide had reduced surface hydrophobicity, and the authors suggested that the highly hydrophobic surface of *S. marcescencs* contributes to its dispersal in the environment and virulence.

Lipopeptide surfactants, such as surfactin from *Bacillus* species and syringomycins from *Pseudomonas* species can act as hemolysins [32], [33], [34], [35]. Serramic acid, another *S. marcescens* product was shown to be hemolytic to human and horse red blood cells, but only poorly hemolytic to bovine and sheep red blood cells [36]. This same study tested serratamolide for hemolytic activity against human red blood cells, and the result was negative. The differences between this current study and the previously described study, in which serratamolide was tested for hemolysis [36], may be due to experimental differences, in that the previous authors delivered serratamolide using liposomes composed of several phospholipids, rather than serratamolide alone. Furthermore, the previous study tested serratamolide against human red blood cells but not sheep or murine red blood cells; it is possible that differences in membrane phospholipid composition or surface proteins may result in differential hemolytic activity against red blood cells from different species, as has been shown before for PhlA [12].

Miyazaki and colleagues showed that serratamolide provided protection to *S. marcescens* against polymorphonuclear leukocyte (PMN) phagocytosis [37]. This is of particular interest because PMNs are the primary leukocyte involved in clearing bacteria corneal infections [38], [39], [40]. Interestingly, it was shown that *Staphylococcus aureus* cells coated with serratamolide were also protected from PMN phagocytosis [37]. This leads us to speculate that the presence of *S. marcescens*-derived serratamolide in contact lens cases or on lenses may better enable other pathogenic bacteria to establish ocular infections.

It was noted that *swrW* was found in ∼35% of the tested ocular clinical isolates, and 40% of the *swrW* containing isolates were hemolysis positive on blood agar plates, suggesting that hemolytic strains express *swrW* sufficiently to produce hemolysis. In support of this premise, mutation of *swrW* in three out of five hemolysis positive strains severely reduced or eliminated hemolysis zones on blood agar plates. Of the *swrW* negative strains, 42% were hemolysis positive, indicating that other mechanisms of hemolysis are present in ocular clinical isolates. Another gene, *swrA* [26], present in some strains of *S. marcescens* is necessary for production of serrawettin W2, may account for the hemolysis positive phenotype of *swrW* negative strains. There is genetic evidence that the *swrA*-dependent product serrawettin W2, a structurally distinct surfactant, can act as a hemolytic agent [41]. Serrawettin W2, consisting of five amino acids with a single acyl chain [25], is detected by *Caenorhabditis elegans* as a chemical signal to avoid *S. marcescens* colonies [41]. Transposon mutation of the *swrA* gene, in strain Db10, led to the loss of hemolysis zones on blood agar plates that was correlated with the loss of serrawettin W2 [41]. Whereas the hemolysis and cytotoxicity data presented here suggest that serratamolide may contribute to bacterial infections, the absence of the *swrW* gene in many pathogenic and contact lens associated strains indicate that SwrW is not a requirement for colonization of contact lenses or for causing ocular diseases. Serratamolide may be more relevant in environmental settings than for human infections, as the majority of pigmented strains tested (66.7%, n = 9) had the *swrW* gene, and pigmentation is generally associated with environmental isolates, whereas clinical isolates are almost exclusively non-pigmented [42]. In an environmental setting serratamolide could contribute to the competitiveness of *S. marcescens* as it is a broad spectrum antibiotic. Furthermore, it was shown that a surfactant produced by *Serratia* sp. ATCC 39006 facilitates the dispersal of the antibiotic pigment prodigiosin [43], and serratamolide may act in an analogous fashion.

Serratamolide has shown promise as an anticancer agent for its proapoptotic effect upon breast cancer and B-cell chronic lymphocytic leukemia cells [44], [45]. Therefore, understanding the pathways that control serratamolide production may yield improved ways to generate this cyclodepsipeptide.

Further studies will focus on determining the regulatory pathway by which CRP regulates serratamolide production, and characterizing the role this surfactant plays in host-pathogen interactions.

## Methods

### Growth Conditions and Strains

All bacteria were cultured with LB medium (per liter: tryptone –10 g, yeast extract –5 g, NaCl –5 g, with or without agar –15 g), except when tested for hemolytic zones on blood agar plates (TSA+5% sheep blood, Becton Dickenson BBL TSA II). Swarming plates consisted of LB medium with 0.6% agar (w/v). Antibiotics were supplemented when needed, with kanamycin at 50–100 µg/ml, gentamicin at 10 µg/ml, and tetracycline at 10 µg/ml. The *Escherichia coli* strains, SM10 and S17–1, were used for conjugations, and EC100D (Epicentre) was used for plasmid preparations. Strains are listed in Table S1. *S. marcescens* strains used were CMS376 (Presque Isle Cultures strain number 3611) [18], Nima, a strain used by pioneering prodigiosin researcher, Robert Williams and colleagues [46], CHASM a compost heap acquired *S. marcescens* isolate [14], and a number of ocular clinical isolates from keratitis patients from the Charles T. Campbell Laboratory of Ophthalmic Microbiology at the University of Pittsburgh Vision Center.

### Genetic Analysis and Plasmids

Transposon mutations were introduced into *S. marcescens* using transposon delivery vectors pBT20 [47] and pSC189 [48], and subsequently mapped, as previously described [18]. Transposon mutant strains were collected in 96 well plates, transferred onto blood agar plates, incubated for 2–3 days at 30°C, and screened for colonies with altered hemolysis zones.

For plasmid generation, chromosomal DNA from CMS376 (Table S1) was amplified using Phusion polymerase (NEB). PCR generated amplicons were mixed with linearized vector DNA in an approximately 10∶1 ratio and used to transform *Saccharomyces cerevisiae* strain InvSc1 with selection for uracil prototrophy, as previously described [49], [50]. Plasmids were isolated from yeast colonies after 3–4 days of growth on uracil-drop out medium [49], and isolated by the smash and grab method [49]. Plasmids were moved into *E. coli* strain EC100D by electroporation, screened by diagnostic PCR, and inserts were verified by sequencing at the University of Pittsburgh Genomics and Proteomics Core facility.

For complementation analysis and overexpression of serratamolide, the *swrW* open reading frame (ORF) was amplified with primers 1630–1631, and cloned using yeast *in vivo* recombineering into pMQ125 that had been linearized with EcoR1, using previously described methods [50]. The pMQ125 vector has an arabinose-inducible promoter and a p15a-based replicon [28].

For insertional mutagenesis of *hexS*, *phlA*, *prtS*, *shlA*, and *swrW*, an internal fragment of each gene was amplified and cloned in either pMQ118 [28] or pStvZ3 [14] that had been linearized with BamH1 using *in vivo* cloning methods as noted above. The primer sets to amplify the internal fragments are listed in Table S2 and were 2014–2015 for *hexS*, 1456–1457 for *phlA*, 996–997 for *prtS*, 1022–1023 for *shlA*, and 1639–1640 for *swrW*. The resulting plasmids were verified using PCR and sequencing, and introduced into the recipient strain by conjugation and selection for kanamycin (100 µg/ml), as previously described [14]. The *fimC* gene was mutated using a pMQ118-based insertion plasmid, pMQ167, as previously described [17].

### Detection of the *swrW* Gene in *S. marcescens* Isolates

Bacteria from frozen stocks were streaked to single colonies on LB or TSA blood agar plates. DNA was extracted from a single colony using Quick Extract (Epicentre) according to the manufacturers specifications. PCR was performed using standard Taq polymerase (New England Biolabs), and standard conditions using the primers set 1639 and 1640 (Table S2) to detect the *swrW* gene. *S. marcescens* (CMS376) and *Staphylococcus aureus* (MZ100) chromosomal DNA were used as a positive and negative controls respectively. Analysis was performed twice with each primer set and any reproducibly produced amplicon of the expected size for any strain was considered a positive result. A quality control PCR reaction was also performed on each DNA preparation to eliminate false negative results using previously described primers (736 and 737) that amplify the *oxyR* gene [18].

### Analysis of *swrW* Expression

For analysis of *swrW* expression, a 351 base pair region of DNA immediately upstream of the *swrW* open reading frame was fused with the tdtomato derivative of *dsred* [51] in pBBR1-based plasmid, pMQ361, yielding pMQ376. The pMQ361 plasmid was made by digesting pMQ131 with SmaI and mixing with a PCR amplicon containing the *nptII* promoter from pMQ118 (Table S1), amplified with primers 2516 and 2517, and an amplicon containing DNA upstream of *swrW* that had been made with primers 2768–9. This region of DNA contains a predicted promoter, as previously noted [21], [22], [23].

The WT and a Δ*crp* mutant strain [17] bearing pMQ376 were grown overnight at 30°C in LB broth (5 ml) supplemented with kanamycin (100 µg/ml) in 20×150 mm glass test tubes rotated on a TC-7 tissue culture rotor (New Brunswick Scientific), set at speed setting 8. The same strains were grown with the empty vector pMQ131 as a control for background fluorescence. Cultures that had grown to saturation (OD_600_ = ∼5.0), were diluted 1∶100 in the same medium and incubated at 30°C with aeration. At designated time points, samples (0.15 ml) were removed to determine culture density (OD = 600 nm) and fluorescence (excitation filter: 545/40, emission filter: 590/20) using a plate reader (Biotek Synergy 2). Background fluorescence was equivalent for both strains (data not shown). Relative fluorescence units (RFU) were determined by dividing fluorescence by culture optical density. The experiment was repeated on two different days with a highly similar result.

To obtain cells for RNA extraction, cultures were first inoculated into 5 ml of LB medium, then vortexed and diluted 1∶5000 in LB to reduce the inoculum. The diluted cultures were grown overnight in LB medium, subcultured to OD600 = 0.1, grown to OD600 = 0.8, subcultured to OD600 = 0.1 and allowed to grow. Samples were taken for RNA analysis until the cultures reached OD600 = ∼3.5. RNA was isolated from bacteria following the method of Wargo, et al, [52] including the three rounds of DNase treatment. RNA was normalized to 50 ng/µl using DNase-free water and 5 µl was used in each reverse transcriptase (RT) reaction using Superscript III RT (Invitrogen) following the manufacturers specifications. A PCR reaction with a 94°C hold for 60 seconds, followed by 30 cycles of 20 seconds at 94°C, 20 seconds at 55°C, and 30 seconds at 72°C, followed by a 72°C hold for 60 seconds was used to detect the amount of transcript from the 16S rDNA gene as a control to normalize samples and the *swrW* gene. Other controls included a no-reverse transcriptase and no-RNA reactions, and these showed that there was no contaminating DNA (data not shown). The 16S rDNA gene was amplified by 2638 and 2639 using previously described primers [11]. The *swrW* gene was amplified with primers 2917 and 2918. Amplicons were run on 1.5% agarose gels and imaged using a Carestream Gel Logic 212Pro device. The experiment was repeated three times with similar results.

### Hemolysis, Swarming and Surfactant Zone Assays

Hemolysis on blood agar plates was performed by plating ten microliters of bacteria from a liquid culture onto blood agar plates and incubating at 30°C for 48 hours.

For quantitative hemolysis assays, fresh mouse blood from C57BL/6 mice were washed in PBS and suspended in PBS at ∼2×10^5^ red blood cells (RBC) per ml. For the hemolysis assay, 70 µl of RBC suspension was incubated with either 50 µl of DMSO or DMSO containing serratamolide (50 µg/ml in DMSO). RBCs were incubated in sterile _dd_H2O for complete lysis. The cells were incubated for seven minutes in microfuge tubes that were centrifuged for 1 minute (500×g), 100 µl of supernatent was transferred to a microplate and the absorbance was read at 405 nm. The experiment was performed in triplicate on two separate days with similar results.

For the serratamolide assay using blood agar plates, a 5 mm hole was cut with a cork borer (Sigma Aldrich Z165220). One hundred microliters of molten LB agar was added to seal the bottom of the well and allowed to harden. One hundred microliters of DMSO or serratamolide in DMSO (1 mg/ml) was added to the well, and the plate was placed at 30°C for 18–20 hours. The experiment was performed on different days with the same result.

Swarming assays were performed as previously described [13] using LB agar with reduced agar concentration (0.6% w/v). Bacteria were placed in a small spot on the top of the agar using a sterile toothpick. The plate was then incubated at 30°C for 18–20 hours. When DMSO or serratamolide were used, the bacteria was applied to the plate followed by ten microliters of DMSO or serratamolide in DMSO (1 mg/ml) that was placed gently on top of the bacteria with a pipetteman.

Optically clear surfactant zones were measured on swarming agar plates after incubation for ∼20 hours at 30°C. The edge of the surfactant zone and colony were marked and the maximum distance was measured in mm.

### Identification and Purification of Serratamolide

For comparative analysis of secreted metabolites of *S. marcescens* wild-type and mutants strains, 10 ml of overnight culture was pelleted. The supernatant was extracted twice with ethyl acetate (5 ml) and the combined ethyl acetate was evaporated *in vacuo*. The residue was dissolved in 0.5 ml MeOH and an aliquot (20 µL) was analyzed using a Dionex HPLC and a Shimadzu LC-MS. Each analysis was repeated at least twice to ensure the reproducibility. For isolation of serratamolide, 1 L of the *swrW* mutant expressing *swrW* from pMQ125+*swrW* induced with arabinose at 0.2% (v/v) was pelleted and the supernant was extracted with ethyl acetate (1 L). Evaporation of ethyl acetate gave the crude residue that was further purified by a Dionex preparative HPLC. The purity of the isolated serratamolide was ensured by high resolution mass spec (HR-MS) and ^1^H NMR analysis in accordance with previously reported data [45] using our previously published methods [53].

### Cytotoxicity Analysis

Human lung carcinoma cells ATCC CCL-185, American Type Culture Collection (ATCC), Manassas, VA) were maintained in Gibco Medium 199 with 100 units/ml penicillin G, 0.1 mg/ml streptomycin and 0.5 mg/ml gentamicin, 10% fetal bovine serum (FBS), and 5% sodium bicarbonate.

Human corneal-limbal epithelial cells (HCLE) [54] were obtained from Dr. Jes Klarlund with permission from Ilene Gipson, and were grown in Keratinocyte-SFM (serum free medium) with L-Glutamine, supplemented with 25 µg/ml BPE, 0.2 ng/ml EGF, and 1 mM CaCl_2_, without any antibiotics.

To measure cytotoxicity, confluent cell layers were exposed to serratamolide in DMSO or DMSO alone, such that the concentration of DMSO in each well was 5% (v/v) for 2 hours at 37°C in 5% CO_2_. Viability of cell layers was assessed using Alamar Blue (Invitrogen, Camarillo, CA). Cells with only DMSO were used as mock wells to indicate full viability (Mock), while triton X-100 (0.25% v/v) was used to determine the reading for non-viable cells (Lysis). After 2 hours, media was removed from all wells, and 200 µl of a 4% Alamar Blue solution in growth medium was added to each well. The plate was returned to the incubator for 1.5 hours at 37°C in 5% CO_2_, and relative fluorescence units (RFU) were determined using a plate reader (Biotek Synergy 2) with a 500/27 excitation filter and a 620/40 emission filter.

The percent cytotoxicity value was determined using RFU values from the Alamar Blue analysis. The following formula was used: 100×(Mock RFU – Sample RFU)/(Mock RFU – Lysis RFU).

### Statistical Analysis

All experiments were performed at least twice on different days with reproducible results. Statistical analysis was performed using Prism 5 software and consisted of two-tailed Student’s T-tests of One-way ANOVA with a Tukey post-test, as noted. Significance was set at p<0.05.

## Supporting Information

Table S1 **Strains and plasmids used in this study.**
(DOCX)

Table S2 **Oligonucleotide primers used in this study.**
(DOCX)
