# Supplementary material for: Serratamolide is a Hemolytic Factor Produced by Serratia marcescens
Source: PLoS One. 2012 May 16;7(5):e36398. doi: 10.1371/journal.pone.0036398 (PMC3353980; doi:10.1371/journal.pone.0036398)
Supplement: Table S1 — Strains and plasmids used in this study. (DOCX) [file pone.0036398.s001.docx]

**Table S1. Strains and plasmids used in this study**

| Strain or plasmid | Description | Reference or source |
| --- | --- | --- |
| InvSc1 | *S. cerevisiae* diploid uracil auxotroph *ura3-52 / ura3-52* | Invitrogen |
| SM10 | *E. coli* conjugation strain *pir* | [17] |
| S17-1 | *E. coli* conjugation strain *pir* | [17] |
| EC100D | *E. coli* cloning strain *pir-116* | Epicentre |
| CMS376 | WT, wild-type strain, Presque Isle Cultures strain number 3611 | [18] |
| CMS524 | *cyaA-2* transposon null mutation | [17] |
| CMS613 | *crp-1* insertion null mutation | [17] |
| CMS786 | *crp-23* transposon null mutation | [17] |
| CMS1687 | *crp-∆4* deletion null mutation | [14] |
| CMS794 | *crp-1 pigB::tn* | [17] |
| CMS1188 | *crp-23 shlA*::pMQ118 | this study |
| CMS3146 | *crp-23* *phlA*::pMQ118 | this study |
| CMS641 | *crp-1 fimC::*tn | [17] |
| CMS627 | *crp-1 swrW::*tn | this study |
| CMS635 | *swrW::*tn | this study |
| CMS2210 | *hexS*::pStvZ3 | this study |
| CMS2868 | *swrW*::pStvZ3 | this study |
| CMS2869 | *crp-∆4 swrW*::pStvZ3 | this study |
| CMS2880 | *hexS*::pStvZ3 *swrW*::tn | this study |
| CMS1787 | Wild-type strain Nima | [46] |
| pMQ118 | suicide vector *nptII, rpsL* | [28] |
| **PLASMID** |  |  |
| pMQ125 | p15a, *P_BAD_*-*lacZa*, pRO1600 | [28] |
| pMQ131 | pBBR1- based shuttle vector | [28] |
| pMQ236 | pMQ118 with and I-SceI site | [28] |
| pMQ240 | pMQ117 + I-SceI meganuclease | [28] |
| pMQ166 | pMQ131 with *crp* ORF | [14] |
| pMQ367 | pMQ125 + *swrW* ORF | this study |
| pMQ215 | pMQ118 with internal *phlA* fragment | this study |
| pMQ163 | pMQ118 with internal *prtS* fragment | this study |
| pMQ187 | pMQ118 with internal *shlA* fragment | this study |
| pMQ361 | pMQ131 with P*nptII*-tdtomato | this study |
| pMQ376 | pMQ361 with P*swrW*-replacing P*nptII* | this study |
| pStvZ3 | oriR6K *lacZ* *nptII* promoter probe | [14] |
| pMQ233 | pStvZ3 with internal *swrW* fragment | this study |

Strains and plasmids used in this study.
