# Supplementary material for: Serratamolide is a Hemolytic Factor Produced by Serratia marcescens
Source: PLoS One. 2012 May 16;7(5):e36398. doi: 10.1371/journal.pone.0036398 (PMC3353980; doi:10.1371/journal.pone.0036398)
Supplement: Table S2 — Oligonucleotide primers used in this study. (DOCX) [file pone.0036398.s002.docx]

**Table S2. Primers used in this study.**

| Primer number | Primer Sequence^a^ |
| --- | --- |
|  |  |
| 736 | tgcaggtcgacGGAGGGAAACAATGAATATTCG |
| 737 | cccggggatccGCTGTCGAGTTGCGCCAGC |
| 996 | cgttgtaaaacgacggccagtgccaagcttgcatgcCCACAACCGGTTACGATGCTG |
| 997 | acaatttcacacaggaaacagctatgaccatgattaGACTGGTTGACGTTGTACCAGG |
| 1022 | gacgttgtaaaacgacggccagtgccaagcttgcatGCATCAACATCATCAACACCG |
| 1023 | gataacaatttcacacaggaaacagctatgaccatgatGCTGGAGGTGTGGTTGCGCTG |
| 1456 | cagctggcgaaagggggatgtgctgcaaggcgattaTCTCAGAACACGCTCAATGCGCAG |
| 1457 | gcggataacaatttcacacaggaaacagctatgaccatgaGCCTTGGCGCTTTTGGCAGC |
| 1630 | atagggcgaattgggtaccgggccccccctcgaggtcgaCGTCAAGGAAGATTGCCGAGC |
| 1631 | aactctctactgtttctccatacccgtaggaggaaaaaTCCGTGTCCGCTTATTCCCTG |
| 1639 | acgacgttgtaaaacgacgggatctatcatcgtggatcctCCTGATTGAAGTTGAACTCC |
| 1640 | gcggtttcccgactggaaagcgggcagtgagcgcGGAGCGCTATAAACAATTAAAAGC |
| 2014 | acgacgttgtaaaacgacgggatctatcataaaaatcctactaCAGAATGGTATCGGCGGTGTCG |
| 2015 | tctagagcggtttcccgactggaaagcgggcagtgagcgcCCCGCGTTCTATAAGCACC |
| 2513 | tctgttttatcagaccgcttctgcgttctgatggatccTTACTTGTACAGCTCGTCCATG |
| 2514 | ctgatcaagagacaggatgaggagaattcATGGTGAGCAAGGGCGAGGAGG |
| 2516 | cctcctcgcccttgctcaccatgaattcTCCTCATCCTGTCTCTTGATCAG |
| 2517 | tgcagctggcacgacaggtttcccgactggagcggccgcACGCTGCCGCAAGCACTCAGG |
| 2638 | AACTGGAGGAAGGTGGGGAT |
| 2639 | AGGAGGTGATCCAACCGCA |
| 2768 | atgaactctttgatgacctcctcgcccttgctcaccatGGAACCACCTCCACAAATGAGG |
| 2769 | gcagctggcacgacaggtttcccgactggacccgggCCACTCCGTTATCGTTCACGCTCC |
| 2917 | CCGTGTGGCTAGACCAATCT |
| 2918 | GAACGAAGGTGTGTTTCTGCC |

^a.^ Sequence in upper case target amplification by PCR. Sequence in lower case target recombination using yeast in vivo cloning.
